# The effect of electronic monitoring combined with weekly feedback and reminders on adherence to inhaled corticosteroids in infants and younger children with asthma: a randomized controlled trial

**DOI:** 10.1186/s13223-020-00466-6

**Published:** 2020-07-29

**Authors:** Jiande Chen, Juan Xu, Liebin Zhao, Jing Zhang, Yong Yin, Fen Zhang

**Affiliations:** Department of Respiratory Medicine, Shanghai Children’s Medical Center, Shanghai Jiao Tong University School of Medicine, No. 1678 Dongfang Road, Pudong, Shanghai, 200127 China

**Keywords:** Asthma, Children, Adherence, Electronic device

## Abstract

**Background:**

Adherence to asthma treatment among children is usually poor. We sought to explore whether electronic adherence monitoring combined with weekly feedback regarding adherence along with a reminder to use inhaled corticosteroids (ICS) would lead to improved compliance with ICS in infants and younger children with asthma.

**Methods:**

96 recruited children (aged 6 months to 3 years) with mild or moderate persistent asthma who were on regular inhaled corticosteroids were randomly allocated to receive electronic monitoring combined with instant messaging software (IMS)-based weekly feedback regarding adherence along with a reminder to keep taking the ICS (intervention group) and to receive electronic monitoring only (control group).

**Results:**

The mean device-monitored adherence was significantly higher in the intervention group (80%) than in the control group (45.9%), with a difference of 34.0% (95% confidence interval [CI], 26.8–41.3%; *P *< 0.001). No difference in the mean caregiver-reported adherence between the interventional group (89.7%) and the control group (92.7%) was observed (*P* = 0.452).

**Conclusions:**

Electronic monitoring combined with IMS-based weekly feedback regarding adherence along with a reminder to keep taking the ICS significantly improved the treatment compliance of infants and younger children with asthma. Caregiver-reported adherence is an unreliable monitoring indicator.

*Trial registration* ClinicalTrials.gov, NCT03277664. Registered 11 September 2017—Retrospectively registered, https://clinicaltrials.gov/ct2/results?cond=&term=NCT03277664

## Background

Daily inhaled corticosteroids (ICS) is associated with improved asthma control, but poor adherence to medication regimens is associated with poor disease control [1], decreased lung function [2], increased need for oral steroids [3, 4], severe attacks of wheeze [5], and increased readmission rate [6]. Adherence of 49–71% of asthma medication has been observed in children and adolescents by objective measurements [7, 8].In infants and young children, adherence to prescribed asthma medication is extremely variable [9, 10] and adherence levels decrease over time [9]: mean adherence in young children (aged 15 months to 5 years) with asthma was 71% in an observational study lasting 2 months [11], but it was only 57% in another study lasting 9 months [12]. Therefore, it’s very necessary to find out effective strategies to improve poor treatment adherence.

Accurately evaluating treatment adherence in children with asthma is the first step to assess the effectiveness of a therapeutic schedule. There are a number of subjective and objective measures for healthcare professionals to assess treatment adherence. However, adherence to ICS monitored by subjective tools such as caregiver report is frequently overestimated compared with objective measures [13]. As an objective method, electronic monitoring devices (EMDs) are regarded as the gold standard in adherence monitoring for their ability to provide detailed information about patterns of treatment use [14].

Effective interventions improving treatment adherence in children with asthma with solid evidence are scarce [8]. Appropriate feedback on treatment adherence by EMD-based reminders [14, 15], sharing adherence data with the child, parent, and physician during the consultation [16], asthma education [17], and real-time medication monitoring with tailored short message service reminders [18] might effectively enhance the treatment adherence among asthma patients.

In this study, we aimed to investigate the effect of electronic monitoring combined with instant messaging software (IMS)-based weekly feedback regarding adherence plus reminder to take medication on adherence to ICS in infants and younger children with asthma.

## Methods

**Fig. 1:**
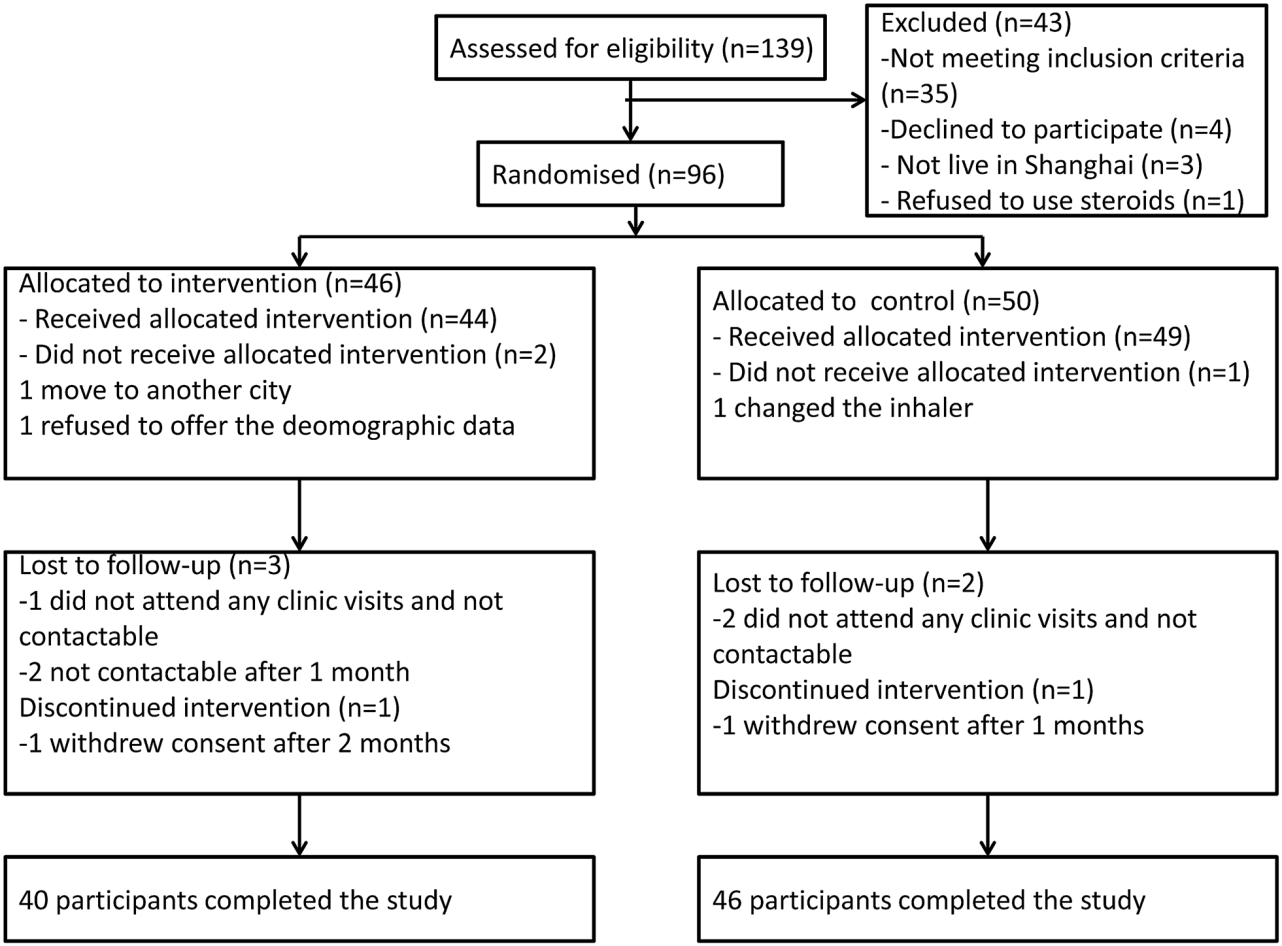
Flow diagram showing progress of participants through trial

This was a multicenter, single-blind, parallel-group randomized controlled clinical trial, with an allocation ratio of 1:1(Fig. 1). Written consent was obtained from the parents of all participants. The study was conducted in accordance with the current Good Clinical Practice, and the protocol was approved by an Independent Ethics Committee or Institutional Review Board for each center. The protocol was registered in ClinicalTrials.gov, number NCT03277664.

### Participants and setting

Children aged 6 months to 3 years who attended Shanghai Children’s Medical Center and 14 community hospitals in Shanghai, China, with doctor-diagnosed asthma were screened for eligibility. Asthma was diagnosed according to Global Initiative for Asthma criteria [19] and to the guideline for the diagnosis and optimal management of asthma in children(2016) [20]. The inclusion criteria were as follows: (1) patients having mild or moderate persistent asthma and (2) patients taking regular ICS with no change in their medication in the last month. At present, budesonide is the only ICS approved by the U.S. Food and Drug Administration for children with asthma aged less than 4 years [20, 21]. The monitoring device available for this trial was compatible only with budesonide nebulizer. Therefore, all participates were prescribed budesonide, and all of them had a nebulizer before recruitment. Participants who had severe persistent asthma or another respiratory disease (eg, a chronic lung disease other than asthma, respiratory health impacted by cardiac conditions, or another medical co-morbidity) or did not live in Shanghai were excluded.

### Interventions

**Fig. 2:**
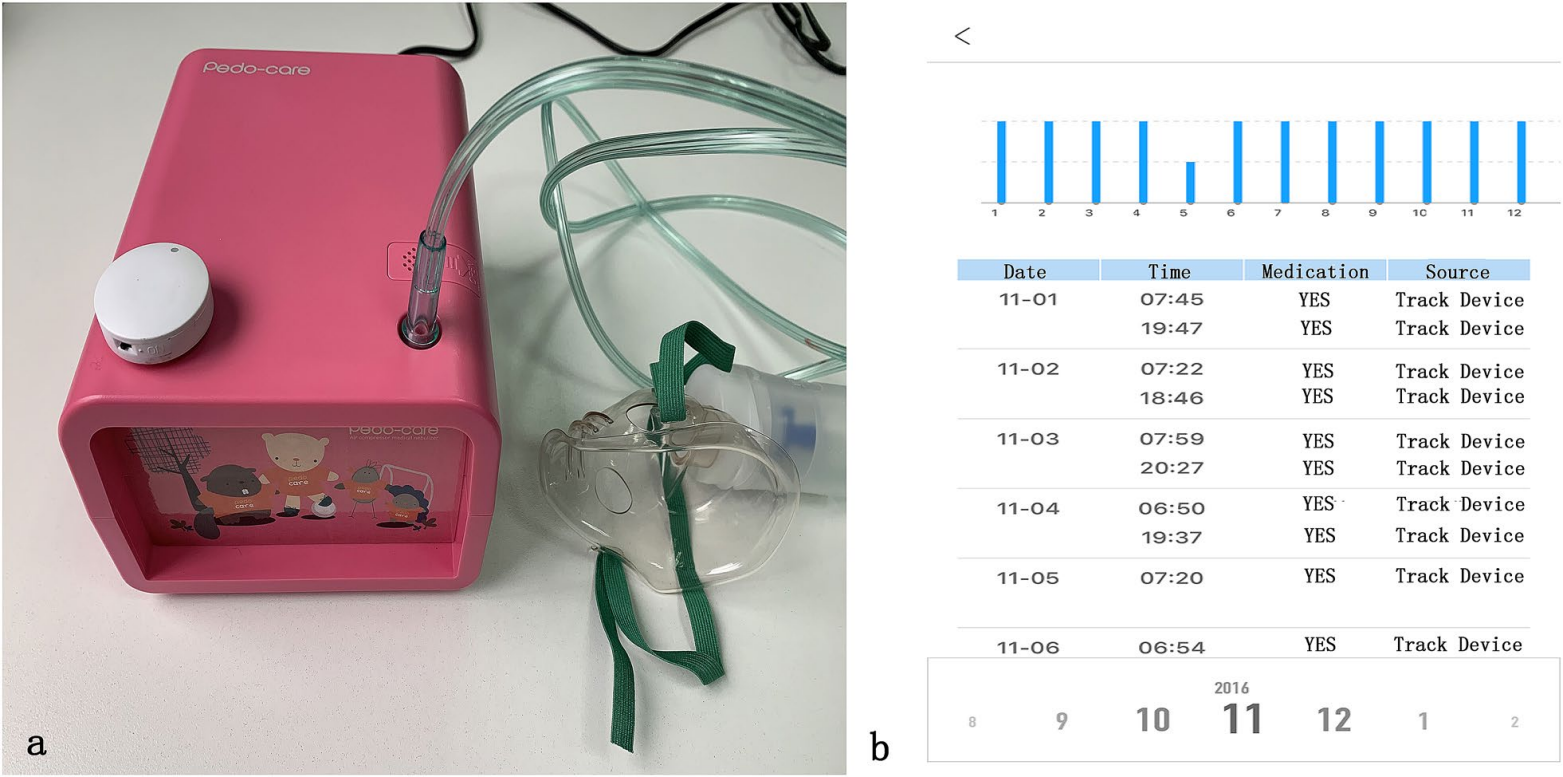
**a** Smart Track Device (white) attached to a nebulizer. The device electronically recorded the date/time of every actuation and automatically sent the usage data to central server via Bluetooth. **b** Example of adherence report from App. The graph shows the number of nebulized medication the patient took each day

Before randomization, all participants had the same chips attached to their regular nebulizers. The SmartTrack Device (Shanghai Sonmol Internet Technology Co. Ltd, Shanghai, CHN; Fig. 2), which is attached to the surface of the nebulizer, can monitor the daily use of the nebulizer. The device records the date, time, and number of actuations used. The usage data were saved in the smart device and automatically transferred to the central server via Bluetooth. All caregivers had their nebulizer technique checked by a qualified asthma nurse and received a brief asthma education session after randomization, emphasizing the importance of taking ICS regularly. All participants were reviewed in their routine asthma clinics 3-monthly, and all treatment decisions were made by the clinical team according to asthma guidelines [19, 20]. Data were collected and adherence rates were calculated weekly.

### Intervention group

All the device-monitored adherence data from the previous week were downloaded from the database and calculated by a qualified asthma nurse. Through free IMS (WeChat; Tencent, Shenzhen, CHN) available on smart phone, the nurse offered feedback to the caregivers weekly according to the adherence rate and reminded them to keep taking the ICS. Caregivers were asked monthly “Has our child inhaled the medicine according to the doctor’s instructions?” and “How about the frequency?” by telephone.

### Control group

All the device-monitored adherence data were downloaded from the background database and calculated weekly. However, feedback and reminders were not given to the caregivers. Caregiver reported medication compliance was assessed monthly, in the same way as the intervention group.

### Primary outcome

The primary outcome of the study was change in the adherence rate monitored by the electronic device for 6 consecutive months. This adherence rate was calculated as the number of device recorded times /number of total times prescribed × 100%.

### Secondary outcomes

The secondary outcomes for the study were the caregiver-reported adherence rate and the difference between device-monitored and caregiver-reported adherence rates for 6 consecutive months. The caregiver-reported adherence rate was recorded on a monthly basis. It was offered by the caregivers of patients through answering the questions “Has our child inhaled the medicine according to the doctor’s instructions” and “How about the frequency”. The device-monitored and caregiver-reported adherence rates were compared monthly.

### Sample size

Tests for two proportions in a repeated measures design were used for sample size calculation by PASS 12 Power Analysis and Sample Size Software (NCSS, LLC. Kaysville, Utah, USA). Group sample sizes of 42 and 42 achieve 80.8% power to detect an odds ratio of 2.679 [22] in a design with 6 repeated measurements having a Compound Symmetry covariance structure when the proportion from group 2 is 0.519 [22], the correlation between observations on the same subject is 0.5, and the alpha level is 0.05. The aim was to recruit 96 participants to allow for a 15% attrition rate.

### Randomization and blinding

Patients were randomly assigned 1:1 to the intervention group or the control group by a researcher using a computer-based minimization procedure who was not otherwise involved in the study. The caregivers of participants and follow-up nurse were not blinded owing to the nature of the intervention. However, the doctors and statisticians were blinded. Adherence data were not available to clinicians in both the groups.

### Statistical analysis

Data were presented as means and standard deviation for continuous variables and frequencies and percentages for categorical variables. In the bivariate analysis, significance was determined by the *t* test for continuous variables and by the Chi square test for categorical variables. General linear models with repeated measures were used to compare adherence levels across time between the groups. A two-tailed *P* value <.05 was taken as statistically significant. Analyses were performed using IBM SPSS Statistics version 25 (IBM, Armonk, NY, USA) software.

## Results

### Patients

From September 2016 to January 2017, 139 asthma patients aged 6 months to 3 years were assessed for eligibility. After exclusions, 96 participants were enrolled and randomly assigned (46 to the intervention group and 50 to the control group). Baseline characteristics were similar in both groups (Table 1). Six-month follow-up was completed for 40 (87.0%) in the intervention group and 46 (92.0%) in the control group; overall, 86 children (89.6%) in total completed the study protocol. No adverse events related to the intervention were identified or reported during the study.

**Table 1:** Characteristics of study participants at randomization

	Intervention group (n = 46)	Control group (n = 50)	*P* value
Age (months), mean (SD)	25.8 (9.6)	27.3 (12.2)	0.501
Male	28 (60.9%)	29 (58.0%)	0.775
Weight (kg), mean (SD)	12.8 (2.1)	13.0 (3.2)	0.806
Height (cm), mean (SD)	88.7 (7.8)	88.4 (9.9)	0.883
First wheeze age (months), mean (SD)	11.9 (8.2)	12.9 (9.6)	0.597
Episodes number of wheezing before the diagnosis of asthma, mean (SD)	5.3 (3.3)	5.5 (3.3)	0.788
Number of ventilator assisted ventilation required before enrollment, mean (SD)	1.1 (0.2)	1.0 (0.2)	0.559
Eczema	25 (54.3%)	26 (52.0%)	0.818
Rhinitis	24 (52.2%)	31 (62.0%)	0.331
Food allergy	14 (30.4%)	16 (32.0%)	0.869
Annual household income (¥ yuan), mean (SD)	212,625.0 (239,205.4)	229,898.0 (252,453.6)	0.743

*SD* standard deviation

### Primary outcome

**Fig. 3:**
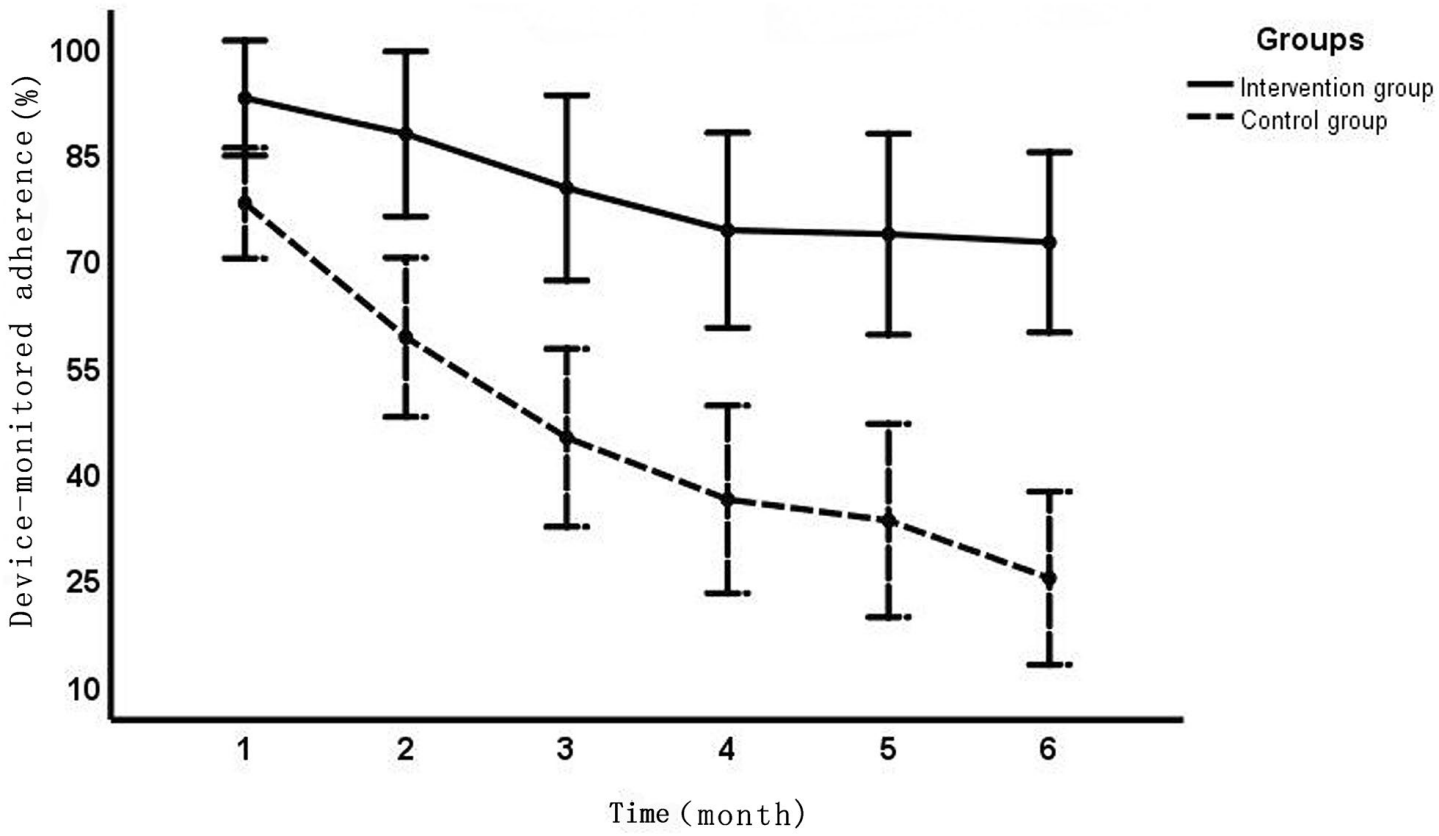
Impact of different interventions and follow-up time on device-monitored adherence

Figure 3 shows impact of intervention (weekly check in and feedback regarding adherence) over time. Compared with the control group (45.9%), the mean device-monitored adherence was significantly higher in the intervention group (80.0%), with a difference of 34.0% (95% confidence interval [CI], 26.8–41.3%; *P *< 0.001). The mean device-monitored adherence difference (95% CI) at 3 and 6 months for the intervention group, compared with the control group was 35.2% (17.3–53.1%) and 47.3% (29.7–64.9%), respectively.

### Secondary outcome

We noted no difference in the mean caregiver-reported adherence rates between the intervention group (89.7%) and the control group (92.7%) (*P* = 0.452). The consistency between the caregiver-reported adherence and the device-monitored adherence was poor (Table 2). The mean caregiver-reported adherence was significantly higher in both groups compared with the mean device-monitored adherence, with a difference of 9.8% (95% CI, 4.0–15.6%; *P* = 0.001) and 46.8% (95% CI, 40.8–52.8%; *P *< 0.001) for the intervention group and the control group, respectively.

**Table 2:** Comparison between caregiver-reported adherence and device-monitored adherence

	First month	Second month	Third month	Fourth month	Fifth month	Sixth month
Intervention group						
Caregiver-reported adherence, mean (SD)	93.0 (20.3)	85.3 (27.9)	92.3 (20.2)	91.1 (23.1)	90.5 (23.1)	86.4 (27.0)
Device-monitored adherence, mean (SD)	92.6 (19.7)	87.5 (28.1)	79.9 (33.5)	74.0 (41.0)	73.4 (42.4)	72.3 (41.5)
*P* value of paired *t* test	0.361	0.35	0.041	0.003	0.024	0.138
Control group						
Caregiver-reported adherence, mean (SD)	95.3 (13.4)	94.9 (14.1)	96.3 (11.4)	92.8 (18.4)	87.6 (24.0)	89.5 (21.5)
Device-monitored adherence, mean (SD)	77.8 (28.7)	58.9 (41.3)	44.8 (44.9)	36.1 (43.0)	33.1 (44.0)	25.0 (36.0)
*P* value of paired *t* test	< 0.001	< 0.001	< 0.001	< 0.001	< 0.001	< 0.001

*SD* standard deviation

## Discussion

To our knowledge, this is the first and largest randomized controlled study powered to detect the effect of electronic monitoring combined with IMS-based weekly feedback and reminders on adherence to ICS in infants and younger children with asthma.

Significantly higher device-monitored adherence observed in the intervention group than in the control group could be caused both by the weekly feedback regarding adherence and by the weekly reminders to keep taking the ICS, since they might effectively address the problem of ‘forgetting’, which has been reported by parents as one of the principal barriers to adherence for young children [23], and improve awareness of non-adherence. However, which part of the intervention is most likely to have caused the study effect is unclear for that we did not set up subgroups to compare the effect of the two on adherence, respectively. It is likely that the weekly reminders to keep taking the ICS in this study played a major role in improved adherence, since using feedback regarding adherence alone in other studies has been observed no improvement in adherence to ICS therapy [17, 24], while using reminders alone was associated with increased adherence to asthma treatment [18, 25].

Similar reminder strategies, such as biweekly telephone education (overall adherence rate: 74.3%) [22], EMD-based audiovisual reminder (median percentage adherence: 84%) [14], and sharing adherence data with the child, parent, and physician during the consultation (mean adherence: 79%) [16] could also significantly increase adherence of asthma treatment. Compared with these methods, ours could achieve comparable results (mean adherence: 80%) and could be considered to be a good alternative for improving adherence to asthma treatment among children. In addition, all of those strategies were mainly implemented among children over 6 years old and may not be suitable for infants and younger children while ours could fill this gap well.

In usual outpatient follow-up process without appropriate intervention, adherence in the control group declined dramatically over the study. This phenomenon may be caused by vary reasons such as “Erratic non-adherence” (forgetfulness or a busy, unpredictable lifestyle), “Unwitting non-adherence” (failure to appreciate the specifics of treatment or the need for adherence), and “Intelligent non-adherence” (a purposeful choice to reduce or discontinue ICS use for many reasons) [26]. However, IMS-based weekly feedback regarding adherence along with a reminder to keep taking the ICS could significantly improve this situation. In the intervention group, adherence fell slightly over time and appeared to be stable after 4 months (over 70%).

Compared with objective measurements, subjective measurements tend to overestimate the level of adherence [7]. In our study, the mean caregiver-reported adherence was also significantly higher in both groups compared with the mean device-monitored adherence. Monthly, we asked caregivers to describe the ICS inhalation frequency prescribed to be taken and to report how many times their child missed taking the ICS in this month. Without appropriate prompts, this kind of caregiver-reported adherence might not be sensitive enough to detect non-adherence. Poor consistency between the caregiver-reported adherence and the device-monitored adherence (Table 2) makes the former not to be a reliable tool for assessing adherence.

One limitation of our study was the lack of investigation on improvements in asthma outcomes among our groups. In fact, similar studies indicate that electronic adherence monitoring with feedback and reminders is likely to be of significant benefit in the improvement of asthma outcomes in children [14, 15]. High levels of adherence to ICS in young children with asthma have been shown to be associated with better asthma control [27, 28] and a reduction in exacerbations [5]. However, in order to make the conclusion more convincing in infants and younger children with asthma, it is recommended that the following related studies should include the evaluation of asthma control levels and exacerbations. The fact that the study was done using nebulized budesonide was another limitation, because this way of administering drugs is much more time-consuming and cumbersome than the metered dose inhaler/spacer combination which is the preferred delivery system for young children with asthma in most of the world. However, each of the delivery devices can be equally efficacious in patients using the correct technique for inhalation [29] and similar devices exist for pressurized metered-dose inhalers. Therefore, our intervention (weekly reminders with information about adherence) can be generalized to other devices/delivery methods.

## Conclusions

These data indicate that electronic monitoring combined with IMS-based weekly feedback regarding adherence along with a reminder to keep taking the ICS significantly improved adherence to ICS in infants and younger children with asthma. Caregiver-reported adherence is not a reliable tool for assessing adherence.

## Data Availability

Raw data for this study were collected on excel sheet using a data pro forma. The data are available from the corresponding author upon reasonable request.

## Abbreviations

CI: Confidence interval
EMD: Electronic monitoring device
ICS: Inhaled corticosteroids
IMS: Instant messaging software
